# Mapping of the exterior architecture of the mesocephalic canine brain

**DOI:** 10.1038/s41598-024-67343-9

**Published:** 2024-07-26

**Authors:** Ahmad Al Aiyan, Rinsha Balan, Even Ghebrehiwot, Yotam Mihreteab, Simona Zerom, Senit Gebreigziabiher, Adnan AlDarwich, Arve Lee Willingham, Uday Kishore

**Affiliations:** Department of Veterinary Medicine, College of Agriculture and Veterinary Medicine, United Arab Emirates University, Al Ain, United Arab Emirates

**Keywords:** Canine brain, Gyri, Sulci, Brain mapping, Neuroanatomy, Neuroscience, Zoology, Anatomy

## Abstract

Despite extensive studies published on the canine brain, inconsistencies and disagreements in the nomenclature and representation of various cerebral structures continue to exist. This study aimed to create a comprehensive mapping of the external architecture of the mesocephalic canine brain with a focus on the major gyri and sulci. Standardized dissection techniques were used on 20 ethically sourced brains obtained from 6 to 10-year-old dogs that were free of neurological disorders. Distinct gyri and sulci with unique locations and bordering structures were observed. Thus, it was possible to identify the often-ignored subprorean gyrus. In addition, this study was able to illustrate the unique locations and bordering structures of gyri and sulci. The findings can contribute to a consensus among researchers on the canine brain anatomy and assist in clarifying the inconsistencies in cerebral structure representation. Furthermore, the results of this study may hold significant implications for veterinary medicine and neuroscience and serve as a foundation for the development of diagnostic and therapeutic approaches for various neurological diseases in dogs. Our findings offer valuable insights into the unique evolutionary adaptations and specialized behaviors of the canine brain, thereby increasing awareness about the neural structures that enable dogs to demonstrate their unique traits.

## Introduction

The domestic canine has long served as a paradigm for experimental studies on the brain and translational neuroscience. It is a valuable research tool that can be used to understand various human diseases, including hereditary eye disease^1^, epilepsy^2^, dementia^3^, and Alzheimer’s disease^4,5^. Dogs can endure noninvasive experimental methods, such as functional magnetic resonance imaging, without being confined; additionally, they have convergent socio-cognitive skills with humans and are highly trainable, in contrast to rats and primates^6,7^. The gyrencephalic pattern of the canine brain is more similar to that of humans^8^.

Researchers have focused on gaining a better knowledge of the canine brain because of the similarities between humans and dogs in terms of growth, aging, and a few diseases that affect the central nervous system^4,9^. A study by Head^10^ reported that dogs with epilepsy exhibit comparable alterations in brain activity to humans with similar conditions, thus offering crucial insights into the underlying pathogenic mechanisms. Studies involving the canine brain have improved our understanding of social behavior and speech, suggesting that dogs can detect and respond to human emotions^11^. These findings provide valuable insights into the behavior and health of animals and humans alike^12^. Advancements in our understanding of comparative neuroanatomy have the potential to improve the diagnosis and treatment of neurological conditions in dogs; additionally, it can augment our understanding of the canine brain, its unique anatomical features, and their contribution to the remarkable abilities and behavior of dogs.

Despite the extensive research conducted on the canine brain, there remains a lack of agreement in the nomenclature and representation of various cerebral structures, particularly gyri and sulci. This inconsistency is evident in the literature, where different names are often used to refer to the same structure. For instance, the pseudosylvian fissure, a prominent landmark in the canine brain, is also referred to as the sulcus pseudosylvius^13^, sulcus sylvius^14^, and lateral sulcus^15^. Such discrepancies can lead to confusion and hinder effective communication and collaboration among researchers.

The main aim of this study is to create an anatomical map of the external architecture of the mesocephalic canine brain by providing a detailed topographic representation of the gross anatomy and nomenclature of the major cerebral sulci and gyri. This study focused on delineating the extensions of the sulci and identifying the gyri that were separated or bordered by them. We believe that the outcomes of this study could be valuable to researchers across this field and help reduce the inconsistencies in the existing literature.

## Materials and methods

All animal experiments were approved by the Animal Research Ethics Committee of United Arab Emirates University and conducted in accordance with relevant guidelines and regulations. The reported experiments comply with the ARRIVE guidelines. In our research, we carefully selected dogs from three representative mesocephalic breeds to ensure a comprehensive understanding of the cerebral hemisphere’s architecture. This selection involved 7 Golden Retrievers, 7 Labrador Retrievers, and 6 German Shepherds. These breeds were chosen as they represent a standard head shape, avoiding the extreme variations seen in dolichomorphs and brachymorphs dog breeds. This strategy ensures that our findings provide a balanced representation of the canine cerebral structure. The brains were obtained from dogs (aged 6–10 years) that had died for various reasons and were free from any known neurological or behavioral disorders. The brains were collected from local animal clinics after obtaining permission from their owners. While our study provides insights into the external architecture of the canine brain, it is important to note that our findings are not influenced by parameters such as age and sex.

To study the external architecture of the dog brain, 10% formaldehyde was injected into the right and left common carotid arteries of all studied samples. A large window was created in the dorsal wall of the skull using a surgical saw. The head was then placed in a container containing 10% formaldehyde and stored in a cold room at 5 °C for 2 weeks, a duration considered optimal for properly fixing the brain tissues. The skull was then carefully opened using surgical tools to expose the brain before being extracted. The meninges were removed from the surfaces of the hemispheres to reveal the cerebral gyri and sulci, which were examined with the naked eye and captured using a Sony a7R II camera.

To study the medial surface of the cerebral hemispheres, a surgical blade was used to cut the brain longitudinally through the longitudinal fissure. The two halves of the brain were separated and flattened to allow for better visualization of the medial surfaces. The cerebral gyri and sulci on the medial surface were examined using the same techniques as those used for the lateral and dorsal surfaces.

Finally, Adobe Photoshop was used to process the images and generate the figures. The different cerebral gyri and sulci were colored and labeled using a standardized coloring technique to improve the clarity of the figures. The brains were handled with care throughout the entire process to ensure the accuracy of the results.

## Results

Several distinct gyri and sulci, each with their unique location and bordering structures, were observed. The beginning and end of the sulci were determined, and each of the identified gyri was named.

### Sulci of the canine brain

**Figure 1:**
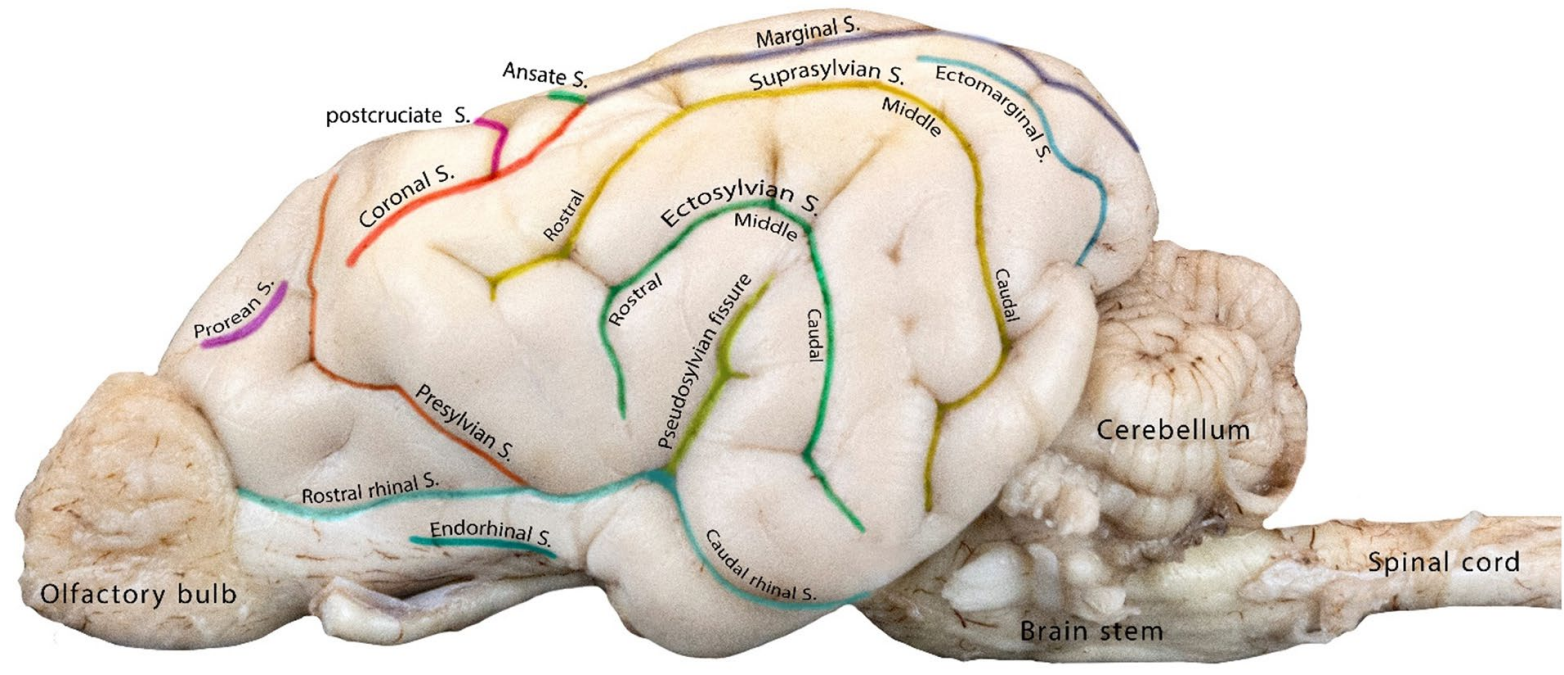
Detailed illustration of the sulci on the lateral surface of the canine brain.

**Figure 2:**
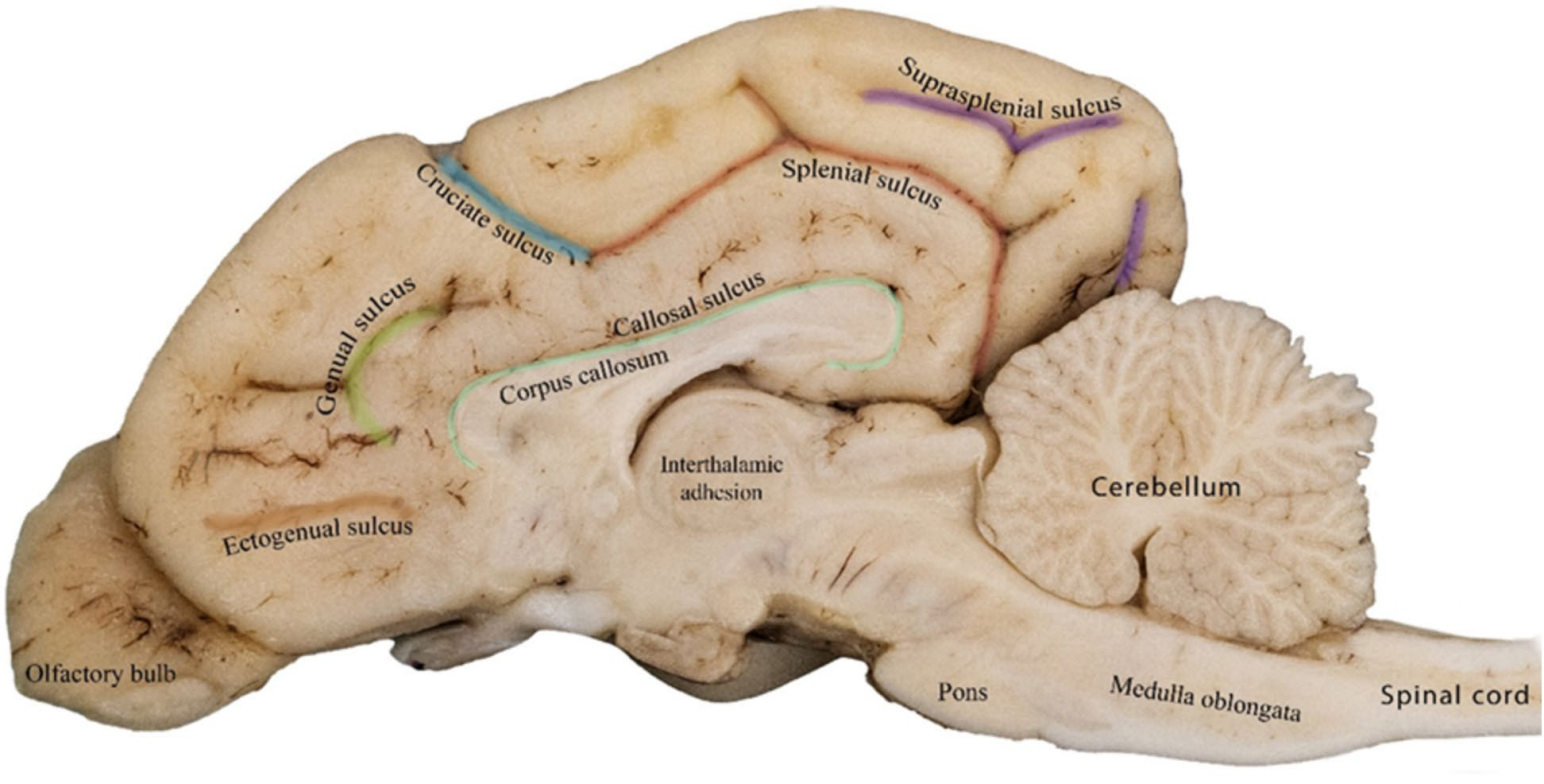
Detailed illustration of the sulci on the medial surface of the canine right cerebral hemisphere.

**Figure 3:**
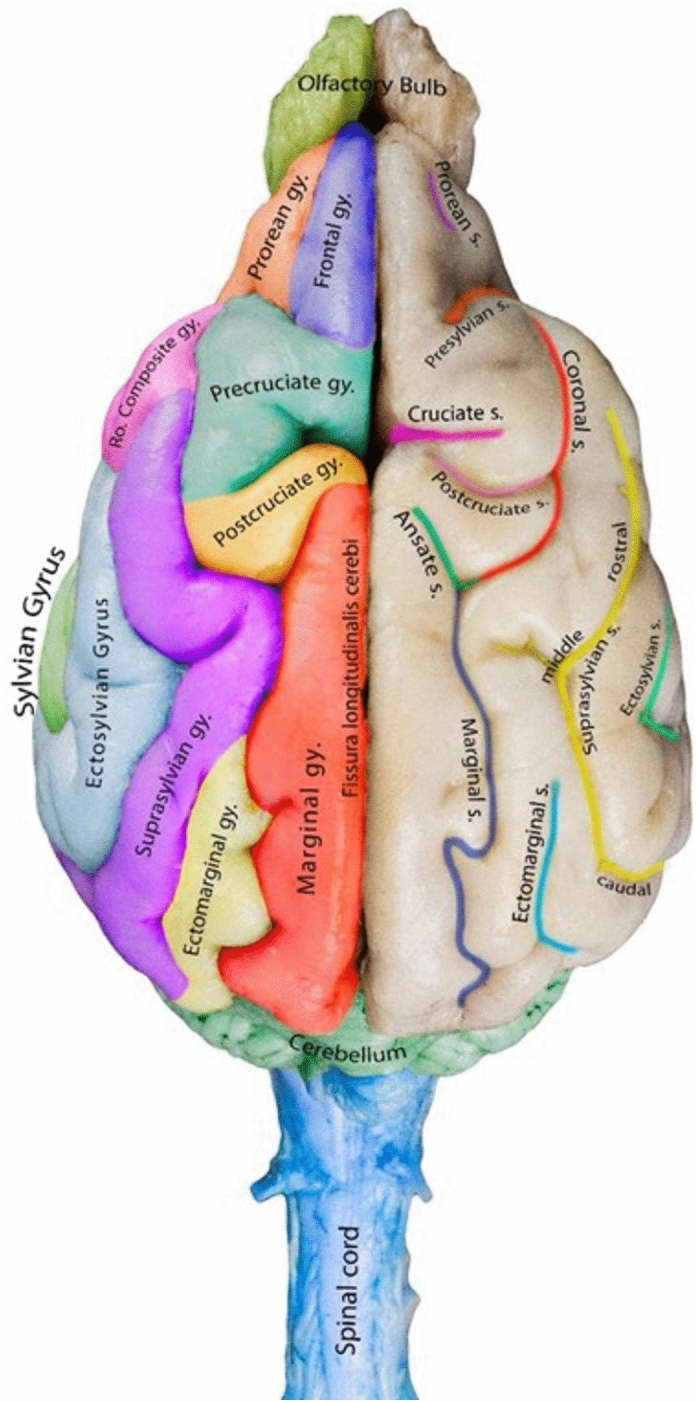
Dorsal view of the canine brain showing the cerebral sulci (right cerebral hemisphere) and the cerebral gyri (left cerebral hemisphere).

Sulci are fissures that define the gyri in the brain and represent extensions of the subarachnoid space. The length and depth of these structures can vary; some are continuous, while others are interrupted (Figs. 1, 2, and 3). The nomenclature of the sulci differs significantly among studies, leading to various interpretations.

#### Pseudosylvian fissure

In this study, the pseudosylvian fissure was identified as the most prevalently recognizable landmark in the lateral hemispheric surface. Moreover, the pseudosylvian fissure was found to divide the lateral rhinal sulcus into the rostral and caudal parts and was entirely enveloped by the Sylvian gyrus (Fig. 1).

#### Presylvian sulcus

The presylvian sulcus was identified as a clear sulcus that formed the caudal border of the prorean gyrus. It separated the prorean and frontal gyri, which are located in front of the sulcus, and the rostral composite and pre-cruciate gyri, which are positioned behind the presylvian sulcus (Figs. 1 and 3).

#### Lateral rhinal sulcus

**Figure 4:**
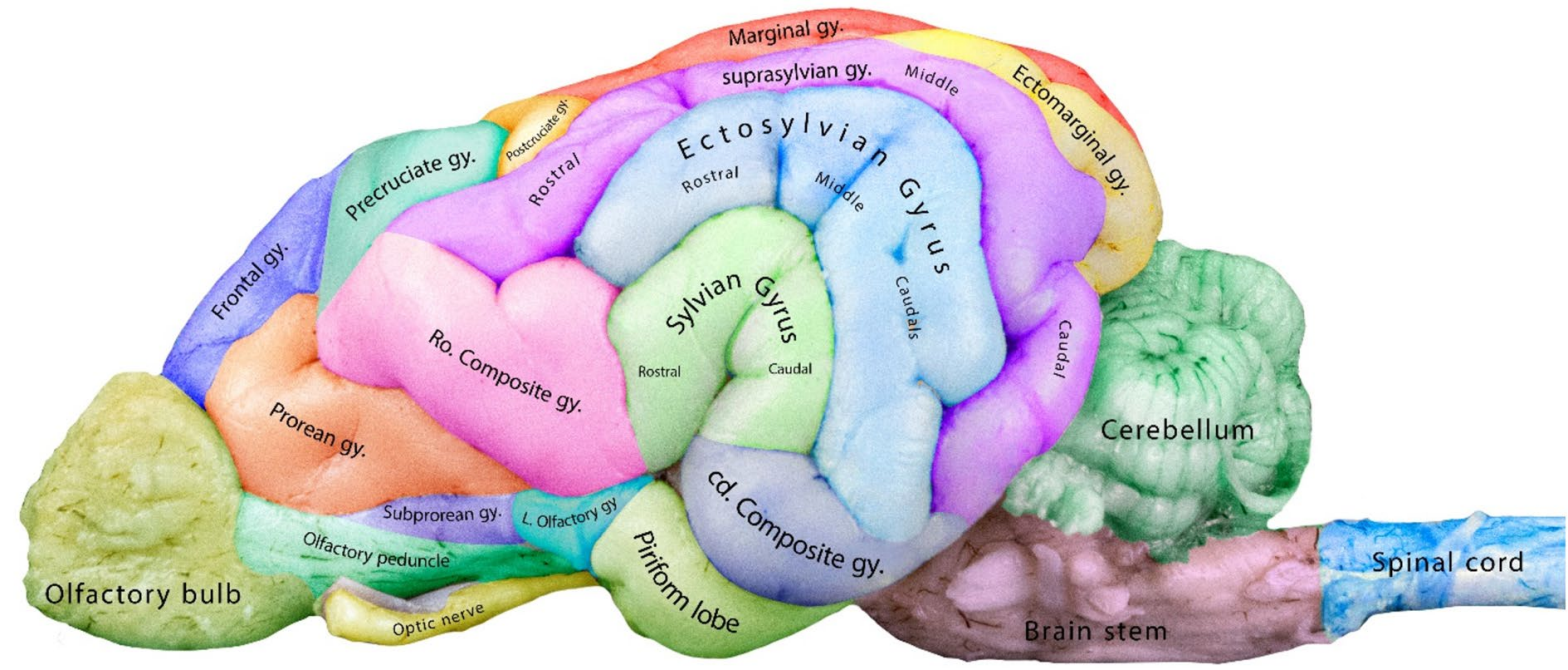
Lateral view of the canine brain showing the cerebral gyri of the left cerebral hemisphere.

The lateral rhinal sulcus lay parallel to the ventral edge of the brain and extended from the caudal end of the olfactory bulb to the caudal end of the cerebrum. It consisted of two parts, the rostral and caudal parts of the lateral rhinal sulcus, which were separated at the point of the pseudosylvian fissure (Fig. 1). The caudal part of the lateral rhinal sulcus presented as a deep groove that separated the piriform lobe, which is the primary olfactory cortex, from the caudal composite gyrus (Figs. 1 and 4).

#### Endorhinal sulcus

The Endorhinal sulcus, observable in the lateral view, was situated between the olfactory peduncle and subprorean gyrus (Figs. 1 and 4).

#### Ectosylvian sulcus

The ectosylvian sulcus was identified as a prominent sulcus that surrounded the Sylvian gyrus from the outside. It had a curved shape with three distinct parts: rostral, middle, and caudal part. The sulcus demonstrated a parabolic pattern in the temporal lobe of the canine brain. It was named the ectosylvian sulcus owing to its location on the lateral surface of the brain, adjacent to the ectosylvian gyrus. The sulcus separated the Sylvian gyrus from the dorsal adjacent ectosylvian gyrus. Furthermore, this sulcus could be seen from the lateral view and partially from the dorsal view (Figs. 1 and 3).

#### Suprasylvian sulcus

The suprasylvian sulcus was identified as a long continuous sulcus that extended dorsolaterally. Dorsally, it delineated the border between the ectosylvian and suprasylvian gyri. The suprasylvian sulcus was divided into rostral, middle, and caudal portions. The rostral portion intersected the rostral part of the suprasylvian gyrus and the rostral composite gyrus. This sulcus could be seen from the lateral and dorsal views (Figs. 1 and 3).

#### Ectomarginal sulcus

The ectomarginal sulcus was located dorsally at the caudal third of the brain and lay parallel to the marginal sulcus. It was clearly visible because it served as a boundary on the caudal third of the ectomarginal and middle suprasylvian gyri. From the caudal view, it coincided with the transverse fissure, almost one-third shifted perpendicularly (Figs. 1 and 3).

#### Marginal sulcus

The marginal sulcus ran parallel to the longitudinal fissure on the dorsal side of the brain. It started around the middle dorsal region of the brain and ran caudally until the caudal end of the brain (Fig. 3).

#### Ansate sulcus

The ansate sulcus was a craniomedial extension of the marginal sulcus, bent toward the longitudinal fissure. It had a unique S-shaped or curved appearance, resembling an inverted U or V shape, with a convex portion facing the front of the brain and a concave portion facing the back (Figs. 1 and 3). This sulcus was better viewed from the dorsal aspect of the brain.

#### Coronal sulcus

The coronal sulcus appeared as an extension of the marginal sulcus situated rostrally. It was located between four gyri: the post- and pre-cruciate gyri dorsally and the rostral extension of the suprasylvian gyrus together with the dorsal aspect of the rostral composite gyrus (Figs. 1 and 3).

#### Cruciate sulcus

The cruciate sulcus presented as a small, deep, and distinct sulcus that lay perpendicular to the longitudinal fissure. The pre-cruciate and post-cruciate gyri were located rostral and caudal to this sulcus, respectively. The sulcus could be viewed from the lateral and dorsal aspects of the brain. The sulcus extended medially and separated the pre- and post-cruciate gyri on the medial surface of the cerebral hemisphere (Figs. 1 and 3).

#### Post-cruciate sulcus

The post-cruciate sulcus was a small, U-shaped sulcus found between the post-cruciate and marginal gyri. It extended laterally to the coronal sulcus and could be viewed dorsally only (Fig. 3).

#### Prorean sulcus

The prorean sulcus was a small sulcus located around the frontal region of the brain between the frontal and prorean gyrus (Figs. 1 and 3).

#### Ectogenual sulcus

The ectogenual sulcus separated the genual gyrus from the frontal gyrus in the medial aspect of the canine brain. It usually appeared as a linear structure that extended rostro-caudally (Fig. 2).

#### Genual sulcus

The genual sulcus separated the rostral portion of the cingulate gyrus from the genual gyrus. It had a curved path on the rostromedial aspect of the canine brain (Fig. 2).

#### Splenial sulcus

The splenial sulcus was a ramified sulcus that separated the post-cruciate from the pre-splenial gyrus rostrally and bifurcated into the rostral and caudal rami, thereby separating the cingulate gyrus from the splenial gyrus completely (Fig. 2). It consisted of two discontinuous caudally situated sulci: one running almost parallel to the longitudinal fissure and the other drawn perpendicular to the transverse fissure separating the marginal gyrus from the splenial gyrus (Fig. 2).

### Gyri of the canine brain

#### Sylvian gyrus

The Sylvian gyrus was found on the latero-ventral side of the cerebral cortex and could be entirely seen from the lateral side of the brain. It was more compact and spread, unlike the suprasylvian gyrus. The gyrus was further divided into the rostral and caudal portions (Figs. 3 and 4) and encompassed the Sylvian fissure (Fig. 1). It was bordered by the rostral composite gyrus cranially, the ectosylvian gyrus dorsally, and the caudal composite gyrus caudally (Fig. 4).

#### Ectosylvian gyrus

The ectosylvian gyrus was located dorsal to the Sylvian gyrus. It was divided into the rostral, middle, and caudal parts, like the Sylvian gyrus. The ectosylvian gyrus was bordered by the rostral part of the suprasylvian gyrus rostrally, rostral composite gyrus rostroventrally, suprasylvian gyrus dorsally, and Sylvian and caudal composite gyrus caudally (Figs. 3 and 4).

#### Suprasylvian gyrus

The suprasylvian gyrus in the canine brain was thin. It extended rostro-caudally from the rostral composite gyrus to the caudal composite gyrus. It consisted of rostral, middle, and caudal portions. It extended dorsally all over the ectosylvian gyrus and ventrally to the pre-cruciate, post-cruciate, marginal, and ectomarginal gyrus (Figs. 3 and 4).

#### Ectomarginal gyrus

The ectomarginal gyrus was located between the marginal and caudal marginal gyri. It was located caudo-dorsally and bordered the suprasylvian gyrus (middle and caudal portions) and the cerebellum ventrally. The ectomarginal gyrus was seen within the occipital cortex (Figs. 3 and 4).

#### Marginal gyrus

**Figure 5:**
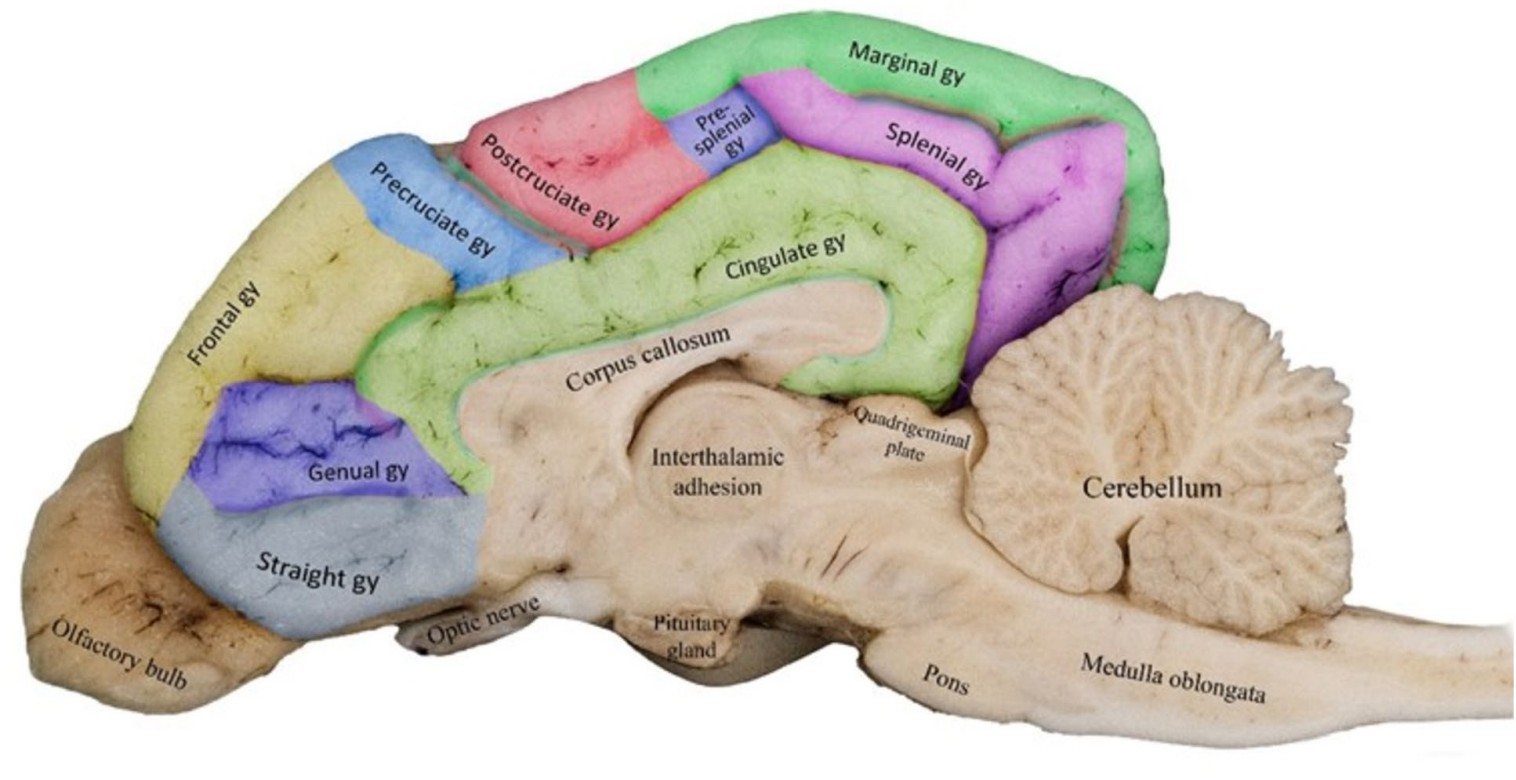
Medial view of the canine brain showing the cerebral gyri of the right cerebral hemisphere.

The dorsal view offered the best visibility of the marginal gyrus, which emerged as the most dorsal structure when observed from the lateral aspect of the brain. It was located above the suprasylvian gyrus, mainly above the rostral and middle portions of the suprasylvian gyrus. It was bordered by the post-cruciate gyrus rostrally and the ectomarginal gyrus caudally. Part of the marginal gyrus could be seen caudally, adjacent to the ectomarginal gyrus, and this portion of the marginal gyrus was referred to as the caudal marginal gyrus. Additionally, it extended to the medial aspect of the cerebral cortex in the outermost dorso-caudal portion of the canine brain (Figs. 3, 4, and 5).

#### Post-cruciate gyrus

The post-cruciate gyrus was located dorsal to the rostral part of the suprasylvian gyrus and rostral to the marginal gyrus. This gyrus was a part of the parietal cortex and was located behind the cruciate sulcus, which separated the frontal and parietal cortex (Figs. 3 and 4).

#### Pre-cruciate gyrus

The pre-cruciate gyrus was located between the frontal and post-cruciate gyrus. Ventrally, it was bordered by the rostral composite gyrus and the rostral part of the suprasylvian gyrus. It was separated from the frontal gyrus and post-cruciate sulcus via the suprasylvian and cruciate sulcus, respectively (Figs. 3 and 4).

#### Frontal gyrus

The frontal gyrus was located within the prefrontal cortex, dorsal to the prorean gyrus and rostral to the pre-cruciate gyrus; it stretched its ramus medially between the olfactory bulb and the pre-cruciate gyrus (Figs. 3 and 4).

#### Prorean gyrus

The prorean gyrus was one of the rostrally located gyri bordered by the frontal gyrus dorsally, the olfactory bulb rostrally, the sub-prorean gyrus and olfactory peduncles ventrally, and the rostral composite gyrus caudally (Figs. 3 and 4).

#### Subprorean gyrus

The subprorean gyrus was found below or ventral to the prorean gyrus. This small gyrus stretched horizontally and was located dorsal to the olfactory peduncle and rostral to the lateral olfactory gyrus (Fig. 4).

#### Rostral composite gyrus

As a central structure, the rostral composite gyrus in the canine brain was surrounded by numerous gyri. It was bordered by the prorean and rostral suprasylvian gyri rostrally, the lateral olfactory gyrus ventrally, and the rostral ectosylvian and Sylvian gyri caudally (Figs. 3 and 4).

#### Caudal composite gyrus

The caudal composite gyrus was located latero-ventrally, dorsal to the piriform lobe, and ventral to the caudal Sylvian and ectosylvian gyrus (Fig. 4). It was located within the ventral temporal cortex.

#### Lateral olfactory gyrus

Our study has revealed that the lateral olfactory gyrus was located lateral to the lateral olfactory tract (Fig. 4). It is also known as the para-olfactory gyrus.

#### Cingulate gyrus

The cingulate gyrus was the innermost medial gyrus located just above the corpus callosum. It extended rostro-caudally and bordered all the medial gyri, except the marginal gyrus (Fig. 5).

#### Straight gyrus

The straight gyrus, also known as gyrus rectus, was positioned on the medial aspect of the frontal lobe, caudodorsal to the olfactory bulb, and ventral to the genual gyrus (Fig. 5).

#### Genual gyrus

The genual gyrus was located at the medial aspect of the frontal lobe of the canine brain; it was bounded by the frontal gyrus dorsally, straight gyrus ventrally, and cingulate gyrus caudally (Fig. 5).

#### Presplenial gyrus

The presplenial gyrus was small, quadrilateral-shaped, and located within the medial aspect of the parietal cortex adjacent to the splenium of the corpus callosum (Fig. 5). It was connected to other medially located gyri, such as the post-cruciate, cingulate, marginal, and splenial gyri.

#### Splenial gyrus

The splenial gyrus was located in the medial aspect of the caudal third of the cerebral cortex, caudodorsally extending from the parietal lobe to the occipital lobe. It was found beneath the marginal gyrus and above the cingulate gyrus.

## Discussion

This study on the nomenclature and representation of canine cerebral sulci and gyri sought to address a significant issue that persists in the field of veterinary neuroscience. This study aimed to provide an extensive topographic representation of the gross anatomy and nomenclature of the mesocephalic canine brain from a variety of perspectives, including lateral, medial, and dorsal views. Since there is some ambiguity regarding the definition of the beginning and end of gyri and sulci, and some inconsistencies and disagreements remain in the nomenclature of gyri and sulci, subjective markings are necessary. To overcome this, we created a colored-labeled mesocephalic canine brain showing the gyri and sulci, as well as their borders. For all gyri and sulci identified in our study, we primarily adhered to the terminology set forth by the Nomina Anatomica Veterinaria^16^ guidelines. Additionally, we expanded our reference base by consulting major anatomy textbooks^17–21^.

Several important characteristics of major sulci and gyri are outlined in this study, and important features were highlighted. The present study revealed that the canine brain has a prominent longitudinal fissure between its two cerebral hemispheres and a rougher medial surface with clearly developed gyri and sulci. The more extensive gyri and sulci may point to a higher level of cortical complexity and processing power, whereas the deeper longitudinal fissure may show a greater degree of functional specialization between the two cerebral hemispheres. In contrast, the ovine brain seems to have a simpler pattern of sulci and gyri than the canine brain^22^. According to Tillet et al.^23^, the ovine brain has a shallow longitudinal fissure and a relatively smooth medial surface.

In dogs, the frontal and temporal lobes overhang the insula bilaterally, forming a prominent sulcus called the pseudosylvian fissure located on the lateral surface. In contrast, Schmidt et al.^24^ reported that the pseudosylvian fissure is less well-defined in dogs than in ruminants. Different names in the literature were used to describe the pseudosylvian fissure, i.e., lateral Sylvian fissure^25^, sulcus pseudosylvius^13^, sulcus sylvius^14^, and lateral sulcus^15^. The pseudosylvian fissure is the base around which three arched gyri (the Sylvian, ectosylvian, and suprasylvian gyri) are arranged, each separated by a corresponding sulcus.

Although the ectosylvian and suprasylvian sulci are present and well-studied in numerous breeds of dogs, their prominence varies depending on their location and how they are depicted in the anatomical presentation^26^. In ruminants, the ectosylvian sulcus had a non-continuous course, which was divided into rostral and caudal parts by the pseudosylvian fissure^24^. In our study, the ectosylvian sulcus in dogs was identified as a prominent sulcus that bordered the Sylvian gyrus from the outside, had a curved shape with three distinct parts: rostral, middle, and caudal parts, and demonstrated a parabolic pattern in the temporal lobe of the canine brain.

The suprasylvian sulcus in the canine brain is an important landmark because it functions as a separation line between the dorsal and lateral hemispheric surfaces of the canine brain. It may be considered a prominent fissure in the camelid brain because it runs through the three limbs, extending from the caudal end of the coronal gyrus to the rostral part of the occipital gyrus^15,27^. In the present study, the lateral rhinal sulcus was subdivided into the rostral and caudal parts, which were located on the lateroventral surface of the hemisphere. The caudal lateral rhinal sulcus was found to be a shallow groove that serves as an important demarcation line for the piriform lobe (Fig. 1).

On the medial surface, the splenial sulcus was the most extensive and deepest sulcus, extending dorsally from the occipital pole and then rostrally around the cingulate gyrus, continuing on the dorsal surface to become the cruciate sulcus. The current findings are consistent with those reported by Datta et al.^28^ regarding the position and extension of the splenial and suprasplenial sulci. Whereas in calves, the splenial sulcus frequently joins the genual sulcus, and in sheep and goats, it terminates on the dorsal surface of the hemisphere^24^.

The ectogenual sulcus was identified as an extension of the genual sulcus, which separated the genual gyrus from the straight gyrus (Fig. 5). This finding differs from those reported in other studies that mainly focused on the anatomical representation of the genual sulcus on the rostral medial aspect of the frontal lobe^8^. In the present study, we successfully identified a previously unrecognized segmental and rostral extension of the genual sulcus, which we have distinguished as a separate feature named the ectogenual sulcus.

In our study, the cruciate sulcus was distinctly deep and easy to identify as it extended from the medial to dorsolateral direction, displacing the surrounding coronal gyrus laterally on each side, resulting in the formation of a cruciate pattern when viewed from a dorsal view. In ruminants, the ansate sulcus, extending from the longitudinal fissure to the dorsal surface and merging with the coronal sulcus, resembled the cross-like arrangement seen in the dog^24^. The continuity usually observed between the ansate and coronal sulci was not found in the case of the cruciate sulcus^24^. The ansate sulcus had a distinct S-shaped appearance in the canine brain. We observed that this sulcus appears to be a rostromedial expansion of the marginal sulcus in dogs. The precise anatomical presentation of the prorean, coronal, and marginal sulci, as discovered in our study, was overlooked in several studies due to its independence from the marginal sulcus. The marginal sulcus demonstrated a longitudinal pattern in dogs, extending up to the caudal border while following a straight path. In camels, however, it shows a wavy pattern, arbitrarily starting from the median position of the hemisphere and ending at the occipital lobe^15^.

The canine brain has been the subject of numerous studies. However, disagreements persist regarding nomenclature and representation of cerebral structures, especially those pertaining to gyri and sulci. The names of these structures vary between publications, with some structures presenting multiple names. For example, in some articles, the gyrus suprasylvius^19^ is also known as the gyrus ectomarginalis or gyrus ectosagittalis^18^. The gyrus postcruciatus^17–19^ is also known as the gyrus sygmoideus posterior^29,30^. Other contradictions include referring to the gyrus marginalis^17,19^ as the gyrus lateralis^30^. One reason for these inconsistencies is the absence of information related to distinct borders for specific brain lobes in dogs, unlike in humans, where features like the central sulcus clearly define the boundary between the frontal and parietal lobes^31^. Additionally, variations in sulci patterns or lengths can lead to differences in the surface morphology of canine brains^32^.

In the present study, the label map emphasized the cerebral sulci and gyri but did not include any lobar distinctions due to the varying definitions of brain lobes in different textbooks, making it challenging to create a standardized lobar distinction. For example, some authors^20,33^ include the post-cruciate gyrus in the frontal lobe, while others include it in the parietal lobe^13,17,18,21^. The parieto-occipital and temporo-occipital boundaries are not clearly defined because of variations in how far the occipital lobe is thought to extend in both the rostral and ventral directions^17,20,21,33^.

Our observations regarding the Sylvian, ectosylvian, and suprasylvian gyri agreed with those described by Datta et al.^28^, Louw^34^, and Czeibert et al.^35^. The three gyri shared similar anatomical representations and relative positions across the various dog breeds, ungulates, and camels examined in these studies. In the present study, these structures appeared to follow a stair-like arrangement in dogs. In our study, we found that the marginal, ectomarginal, and suprasylvian gyri extend caudally to form the occipital cortex. The occipital region in the dog brain is relatively narrow compared to other animals, such as the camel, where the gyrus occipitalis is more prominent and clearly separated from surrounding gyri^27^.

There are two dominant gyri arranged side by side in the dorsocaudal region of the hemisphere: the marginal and the ectomarginal gyri. A similar finding was reported by Evans and de Lahunta^19^, Louw^34^, and Czeibert et al.^35^.

The pre-and post-cruciate gyri were larger and located more dorsally in the canine brain compared with the camel brain^15,27,36^. Gerussi et al.^37^ suggested similarities between the post-cruciate gyrus of the canine and ovine brains in terms of somatotopic organization. However, when comparing the equine and canine brains, the former has a less prominent post-cruciate gyrus, which may explain why dogs have faster reaction times and better somatosensory processing than horses^38^.

We observed that the frontal gyrus could be found in both the lateral and medial sides of the canine cerebral hemisphere, and it is situated rostral to the pre-cruciate gyrus, adjacent to the genual gyrus on the medial side, and the prorean gyrus on the lateral side, similar to what was reported by Johnson et al.^8^. In the camel brain, the frontal gyrus is not present^15,27,39^.

Consistent with the findings reported by Czeibert et al.^35^ and Andrews et al.^40^, the rostral and caudal composite gyri were located on the lateral hemispheric side. They were named the rostral and caudal composite gyri based on their position relative to the Sylvian and ectosylvian sulci in the canine brain. These two gyri are absent in the camel brain^15,27^.

One of the most striking features noticed in the present study was a small vertically stretched gyrus located ventral to the prorean gyrus, dorsal to the olfactory peduncle, and rostral to the lateral olfactory gyrus. This gyrus, called the subprorean gyrus, is often an overlooked segment of the canine brain^28,35^. However, Johnson et al.^8^ noted it in their study of the canine brain. We were able to locate this segment successfully in all the studied samples.

The lateral olfactory gyrus is relatively prominent and easy to identify in the latero-medial part of the canine brain^41^. The heightened significance of olfaction in the survival and perception of dogs has led to the development of more advanced olfactory pathways in their brains^42^. We anticipate that conducting further research on the cerebral arterial branches in dogs, similar to previous studies on other animals^43,44^, will advance our comprehension and identification of the cerebral sulci and gyri.

In our study, we focused on mesocephalic dog breeds while excluding both brachycephalic and dolichocephalic breeds. This helped us to minimize variations in brain shape within our template and ensure greater consistency. It should be noted that our findings are not influenced by factors such as age, sex, or breed-specific variations.

## Conclusions

In conclusion, this study thoroughly analyzes the distinctive anatomical features of the mesocephalic canine brain. In addition to helping veterinary surgeons pinpoint the precise gyri and sulci during canine brain surgery, the findings of this study may aid in reducing the inconsistencies and discrepancies among various studies regarding the gross anatomy of the canine brain. Furthermore, this study may assist in clarifying the variations and disagreements about the nomenclature and representation of the canine cerebral structures and aid in establishing a common understanding. We believe that these findings are crucial for veterinary medicine, animal behavior, and neuroscience research, as they provide a solid foundation for developing diagnostic and therapeutic approaches for various neurological diseases in dogs.

## Data Availability

The datasets for this study can be made available by the corresponding author, without undue reservation.
